# Field Decorrelation and Isolation Improvement in an MIMO Antenna Using an All-Dielectric Device Based on Transformation Electromagnetics

**DOI:** 10.3390/s21227577

**Published:** 2021-11-15

**Authors:** Usman Qureshi, Muhammad Umar Khan, Mohammad S. Sharawi, Shah Nawaz Burokur, Raj Mittra

**Affiliations:** 1 Research Institute for Microwave and Millimeter-Wave Studies, National University of Sciences and Technology, Islamabad 44000, Pakistan; uqureshi.msee17seecs@seecs.edu.pk; 2 Department of Electrical Engineering, Polytechnique Montréal, Montréal, QC H3T 1J4, Canada; mohammad.sharawi@polymtl.ca; 3 LEME, UPL, Univ Paris Nanterre, F92410 Ville d’Avray, France; sburokur@parisnanterre.fr; 4 Department of Electrical and Computer Engineering, University of Central Florida, Orlando, FL 32816, USA; rajmittra@ieee.org

**Keywords:** correlation coefficient, multiple-input multiple-output (MIMO) antenna, transformation electromagnetics, 3D printing, additive manufacturing

## Abstract

This work presents a new technique for enhancing the performance of a multiple-input multiple-output (MIMO) antenna by improving its correlation coefficient *ρ*. A broadband dielectric structure is designed using the transformation electromagnetics (TE) concept to decorrelate the fields of closely placed radiating elements of an MIMO antenna, thereby decreasing *ρ* and mutual coupling. The desired properties of the broadband dielectric wave tilting structure (DWTS) are determined by using quasi-conformal transformation electromagnetics (QCTE). Next, the permittivity profile of the DWTS is realized by employing air-hole technology, which is based on the effective medium theory, and the DWTS is fabricated using the additive manufacturing (3D printing) technique. The effectiveness of the proposed technique is verified by designing two-element patch-based MIMO antenna prototypes operating at 3 GHz, 5 GHz, and 7 GHz, respectively. The proposed technique helped to reduce the correlation coefficient *ρ* in the range of 37% to 99% in the respective operating bandwidth of each MIMO antenna, thereby, in each case, improving the isolation between antenna elements by better than 3 dB, which is an excellent performance.

## 1. Introduction

The rapid evolution of wireless technology and the continuous demand for higher channel capacities, as well as better link reliabilities, demand a continuous review and upgrade of wireless standards. Multiple-input multiple-output (MIMO) is one such technology, which is constantly finding its way in the current and future wireless standards mainly because it can offer better spectral efficiency and link reliability. MIMO systems use multiple yet independent data streams between antenna elements at both transmitter and receiver ends to meet these requirements within limited bandwidth and power [1]. Wireless communication standards, including WLAN, WiMAX, fourth generation (4G), and fifth generation (5G), rely heavily on MIMO systems. These systems exploit the spatial dimensions of the multipath propagation channels in two different ways, i.e., spatial multiplexing or spatial diversity.

The diversity or multiplexing gain offered by a system depends upon the number of uncorrelated channels formed between the transmitter and receiver. In order to reap the benefits of an MIMO system, high port isolation and low correlation among channels are essential [2]. The degree of correlation among the channels is mathematically represented by a parameter known as the correlation coefficient *ρ*. It is a function of the complex three-dimensional radiation patterns of the MIMO antenna elements, as well as the propagation environment. Normally, an isotropic propagation environment of balanced polarizations is assumed for MIMO system operation, thereby making *ρ* solely a function of the radiation patterns of the antenna elements [3]. Thus, the antenna plays an important role in an MIMO system and, therefore, its design requires particular consideration. *ρ* is an important performance metric of MIMO antenna design, since it determines the degree of correlation that an antenna design will add to an MIMO system, thereby affecting its performance. A low value of *ρ* indicates a good MIMO antenna design, which results in an overall good performance of the MIMO system [2].

Computation of *ρ* is a tedious and expensive task since three-dimensional radiation patterns (both magnitude and phase), in both polarizations, are required for each MIMO antenna element. Hence, a much simpler expression has been proposed, which utilizes the *S*-parameters of the MIMO antennas to evaluate *ρ* [4], providing a direct relationship between the latter coefficient and the port isolation between the antenna elements. This has led to the understanding that the use of isolation enhancement/port decoupling techniques would help to lower the value of *ρ* [5,6,7,8,9]. As a result, subsequent works on MIMO antenna design often focused on similar design strategies. However, later works [10,11] have shown that, in general, the expression for *ρ* based on *S*-parameters is inaccurate and that, consequently, increasing the port isolation may not guarantee a reduction in *ρ*. Nevertheless, high port isolation and low mutual coupling between elements are still necessary to obtain good radiation efficiency and improved signal-to-noise ratio of a propagation channel.

From the original formulation of *ρ* in [10], it is evident that the far-field radiation patterns of the individual antenna elements are the factors that determine *ρ*. Keeping this formulation in perspective, *ρ* can be reduced by adopting one of three different methods: (a) decorrelating the phase of the electric fields of the two elements by placing the elements far apart; (b) decorrelating the magnitude of the electric fields of the two elements by using radiating elements whose beams are tilted in opposite directions; (c) decorrelating in terms of polarization of electric fields of the two elements by aligning the elements orthogonally to each other. According to the second method, a practical approach was proposed in [12], in which a phase gradient partially reflective surface (PRS) was placed above patch-based MIMO antenna elements in a Fabry–Perot cavity configuration. The technique caused the radiation patterns of individual antenna elements to be tilted away from each other, resulting in a considerable reduction in *ρ*. However, the drawbacks of this method are not only the narrowband feature but also the increase in mutual coupling between the antenna elements due to presence of the reflecting surface above them, which is not desirable.

This work presents a new technique for a simultaneous reduction in *ρ* and mutual coupling by following the similar concept of tilting the radiation patterns of each of the elements of a patch-based MIMO antenna away from each other. The concept was first introduced briefly in [13], where an ideal all-dielectric device was numerically simulated. This work further explains the practical realization of the ideal and proposed technique using an all-dielectric polylactic acid (PLA) material. In this work, a broadband all-dielectric device is designed to control the direction of the electromagnetics wave propagation. The device is based on the concept of transformation electromagnetics (TE) and is fabricated using 3D printing. The device reduces the pattern overlap when placed above the antenna elements, operating at 3 GHz, 5 GHz, and 7 GHz, by tilting the beams away from each other, which in turn decreases *ρ*. Moreover, the port isolation is also improved because of the reduction in the field coupling and nonexistence of a cavity configuration. Thus, three design benefits are achieved, as opposed to the configuration proposed in [12], where only *ρ* is reduced at the expense of a decrease in port isolation.

The remainder of the paper is organized as follows: Section 2 describes the TE-based methodology for designing the broadband dielectric structure for wave tilting. The first part of Section 3 presents the design of all three two-element MIMO antennas. In the second and third parts, we respectively present ideal and realistic PLA-based dielectric TE structures, designed by using the guidelines detailed in Section 2. The fourth part presents the complete antenna systems with ideal and PLA-based TE structures. Section 4 presents the simulated and measured S-parameters, radiation patterns, and correlation coefficient results of the fabricated prototypes. Lastly, Section 5 concludes the paper with a summary of the presented study.

## 2. Design Methodology

In order to reduce *ρ*, a simple approach consists of spatially decorrelating the far-field radiation patterns of individual antenna elements within an MIMO antenna system. According to the field-based formulation of *ρ*, this can be achieved by tilting the beams of the individual antenna elements away from each other. Such a beam tilt for individual elements of an MIMO antenna can be obtained by designing an all-dielectric structure which has a varying permittivity profile. The relationship of the permittivity profile of the structure and the required beam tilt is computed by using a systematic methodology of TE.

TE is a space-transformation technique, which is based on the form invariance of Maxwell’s equations under coordinate transformation. This concept is useful for designing devices to control the electromagnetic wave propagation within the material in a desired manner by spatially varying the constitutive parameters of the material [14]. Essentially, TE provides a unique approach to modify the field distribution or to mimic the spatial transformation, by adjusting the variables of Maxwell’s equations [15]. The TE approach involves two essential steps. First, the desired space deformation is achieved by applying a specific coordinate transformation function to control the electromagnetic field distribution in the device. Second, the material parameters of the device are determined to produce the field distribution achieved by the coordinate transformation using the form invariance property of Maxwell’s equations.

For antenna beam tilting, several coordinate transformation-based devices have been reported in the literature [16,17], although the realization of most of them have turned out to be challenging. This is because all of the nine tensor components of the permittivity and permeability of the required material parameters of the transformed medium are both anisotropic and spatially inhomogeneous. Furthermore, the TE approach does not provide any insight into how to physically realize the desired material distribution. Exacerbating the problem is the fact that typically all of the required permittivity and permeability tensors dictated by the TE mapping have extreme ranges of values that are not found in conventional materials. Artificially engineered subwavelength structures of metamaterials have been widely used to achieve such ranges of values, but they limit the operational bandwidth and performance of the TE device because of their highly dispersive nature. Furthermore, their insertion loss is often in the unacceptable range.

To circumvent the problem, the relationship between spatial deformation and coordinate transformation was proposed on the basis of Laplace’s equation [18], as opposed to Maxwell’s equations. If Laplace’s equation is used with appropriate boundary conditions, such as Dirichlet or Neumann, then the achieved mapping is quasi-conformal. It was shown in [19,20] that quasi-conformal transformation electromagnetics (QCTE) mapping can be used to overcome the difficulties with TE mentioned above, enabling the practical realization of TE devices by using purely graded index dielectric materials that are quasi-isotropic, as well as non-resonant. Such a quasi-isotropic profile indicates broad frequency bandwidth operation characteristics.

QCTE mapping is used to design the broadband dielectric-based structure for beam tilting. It first defines the electromagnetic wave propagation in an air-filled virtual domain which represents the desired pattern. This pattern is then transformed to the physical domain, which represents the transformed medium [21]. This medium performs the same operation of electromagnetic wave steering as described in the virtual domain. Constitutive parameters of the obtained medium define the beam steering angle. If the dimensions of the considered excitation source are larger than size of the medium, then the beam deflection will be smaller [22]. The transformed medium obtained from QCTE, after ignoring the in-plane anisotropy in the permittivity tensor, represents a quasi-isotropic graded index dielectric profile in which only one component tensor of permittivity has spatial inhomogeneity.

To achieve the desired objective of low envelope correlation, the beams of each antenna elements are titled away from each other. This is realized by placing the proposed TE structure above the MIMO antenna to create a symmetry at the center point of its radiating elements. The spatially varying distribution of refractive index of the device is introduced along the axis on which the antenna elements reside, such that the beams deflect in opposite directions as the respective waves pass through the structure.

## 3. Proposed Design Details

### 3.1. Radiating Elements

**Figure 1:**
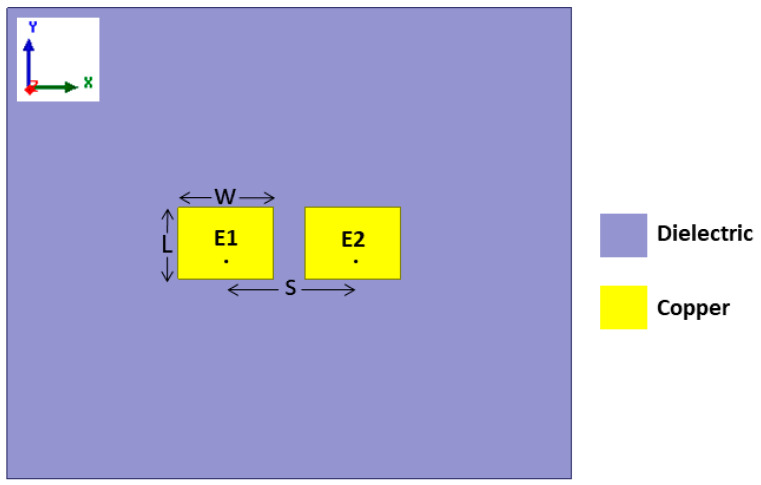
Schematic diagram of the considered two-element MIMO antenna.

To verify the broad frequency bandwidth operation of the proposed DWTS, three different two-element patch-based MIMO antennas operating at 3 GHz, 5 GHz, and 7 GHz were used due to their narrow band frequency response. However, in real-world applications, it is expected to use a radiator with a wideband frequency response. Figure 1 shows a two-element MIMO antenna consisting of two closely spaced identical rectangular patch elements (E1 and E2). These elements were printed on a 1.524 mm thick RO4003 Rogers substrate (*ε*r = 3.55, tan*δ* = 0.0027), with lateral dimensions of 133 × 90 mm^2^. For each frequency, choosing different dimensions of length (*L*) and width (*W*) of both patch elements allowed achieving resonant frequencies of 3 GHz, 5 GHz, and 7 GHz. The *L*/*W* values of both patch antenna elements at 3 GHz, 5 GHz, and 7 GHz were 25.23 mm/30.7 mm, 14.68 mm/18.5 mm, and 10.18 mm/12 mm, respectively. The center-to-center separation (*S*) between the antenna elements was 36.7 mm (0.37λ) at 3 GHz, 24.5 mm (0.41λ) at 5 GHz, and 16.29 mm (0.38λ) at 7 GHz, which resulted in an edge-to-edge separation of 6 mm (0.06λ), 6 mm (0.1λ), and 4.29 mm (0.1λ), respectively. Here, λ represents the free-space wavelength. Each antenna element at their resonance frequencies was excited by a coaxial feed with an offset distance of 4.2 mm, 2.7 mm, and 1.9 mm from the center of patch. The inner diameter of the conducting pin was 1.27 mm, whereas the outer conductor diameter was 4.2 mm, which was used to connect the ground of the antenna to the body of the coaxial connector. The overall length of the coaxial connector considered during numerical simulations was 12 mm.

### 3.2. Dielectric Wave Tilting Structure (DWTS)

**Figure 2:**
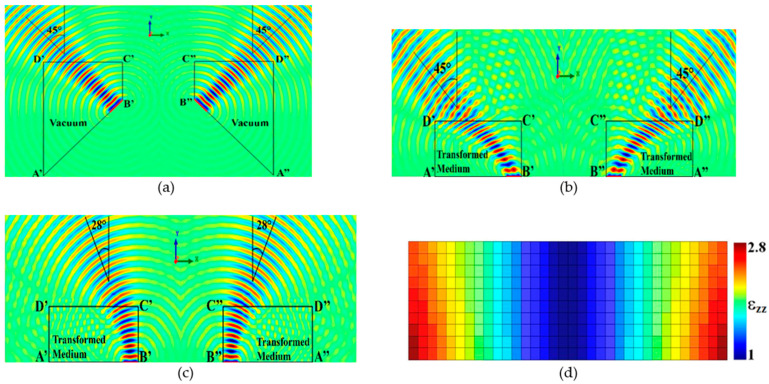
Space mapping illustration. (**a**) Virtual domain. (**b**) Physical domain with anisotropic material parameters. (**c**) Physical domain with simplified quasi-isotropic material parameters. (**d**) DWTS permittivity (*ε_zz_*) profile (*xoz*-plane).

The design of the dielectric wave tilting structure (DWTS) to skew the beams of the two elements was based on the guidelines discussed in Section 2 and the theoretical model detailed in [21]. An air-filled virtual domain was first defined with a wave propagating at a tilt angle of ±45° as shown in Figure 2a. The transformed medium was then obtained using the QCTE mapping, which defined a medium with varying dielectric constant in the physical domain creating the same degree of beam tilt as described in the virtual domain. The transformation matrix was obtained by solving Laplace’s equation using the partial differential equation (PDE) solver of COMSOL Multiphysics subject to predefined Neumann and Dirichlet sliding boundary conditions. The field profile obtained due to the transformed medium is shown in Figure 2b.

The transformed medium obtained to achieve the desired tilt in the beam had continuous variation of the permittivity. Such a medium is difficult to be realized physically. This issue was tackled by converting the inhomogeneous permittivity profile into discrete steps of varying permittivity values. Using the method given in [23], the permittivity profile of the proposed DWTS was varied in discrete steps between 1 and 2.8, representing the transformed medium. As the available dialectic material in our 3D printing facility had a dielectric constant of 2.65, values higher than 2.65 in the discrete permittivity profile were assumed to be equal to the dielectric constant of the PLA. The realized structure comprised 340 (34 × 10) unit cells with a unit cell dimension of 3.5 × 3.5 × 70 mm^3^ each. Figure 2d shows the permittivity value of each unit cell. It is important to note that the conformal module, which is a geometric quantity determined by the structure and containing the complete invariants of the structure, of the physical space was much larger than 1 compared to that of the virtual space, which was 1. As such, anisotropy in the material parameters tensor was introduced in the transformed medium [21]. However, for a possible experimental validation of the proposed DWTS using a simplified all-dielectric material, we propose to ignore the anisotropy in the material parameters tensor. Such a simplification led to a degradation of the beam steering characteristics, which was reduced to around 28° in the case of 3 GHz, as illustrated in Figure 2c.

### 3.3. Realistic Design of the DWTS

According to effective medium theory, an isotropic and homogeneous material can be designed if the operating wavelength is large when compared to size of the unit cell. There exists a tradeoff between the desired bandwidth and the TE structure size for which a homogeneous transformed medium can be designed in any frequency range. At the lower limit of the frequency range, the size of the DWTS must be big since the wavelength is large and, at the upper limit, it is restricted by the small wavelength. Therefore, at high frequencies, the unit cells must be engineered with respect to the wavelength so as to be consistent with the effective medium theory. Furthermore, smaller dimensions of the transformed medium as compared to excitation source result in smaller control of wave propagation in the desired direction [22]. A discrete model is, therefore, proposed in this work for possible physical realization of DWTS using 3D printing additive manufacturing technology.

Generally, PLA-based dielectric material can be used to provide permittivity values greater than 1. In this work, the discrete permittivity profile of the DWTS was realized from non-resonant cells composed of dielectric cubes with air holes with the aim of using the device over a broad frequency range spanning from 3 GHz to 7 GHz. The permittivity value of each unit cell was designed in such a way that its value can be considered homogeneous across the cell. This was achieved by using a unit cell whose size was very small compared to the operating wavelength. The effective permittivity *ε_eff_* of the composite material comprising two materials, i.e., air and dielectric, can be evaluated as follows [19]:

(1) $$\varepsilon_{eff}=\varepsilon_{a}f_{a}+\varepsilon_{h}f_{h},$$

where *f_h_* and *f_a_* are the fractional volumes of the dielectric PLA material and air holes, respectively, and *ε_h_* and *ε_a_* are the relative permittivities of the dielectric and air, respectively.

**Figure 3:**
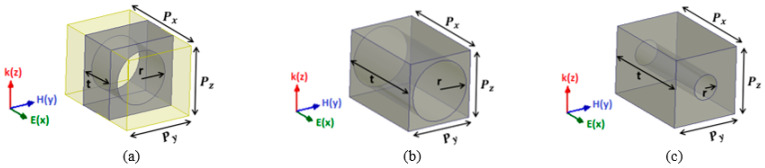
Unit cell of dielectric host material with air hole where *P_x_* = 5 mm, *P_y_* = *P_z_* = 3.5 mm. (**a**) Unit cell for effective permittivity of 1.25. (**b**) Unit cell for effective permittivity of 1.63. (**c**) Unit cell for effective permittivity of 2.5.

**Figure 4:**
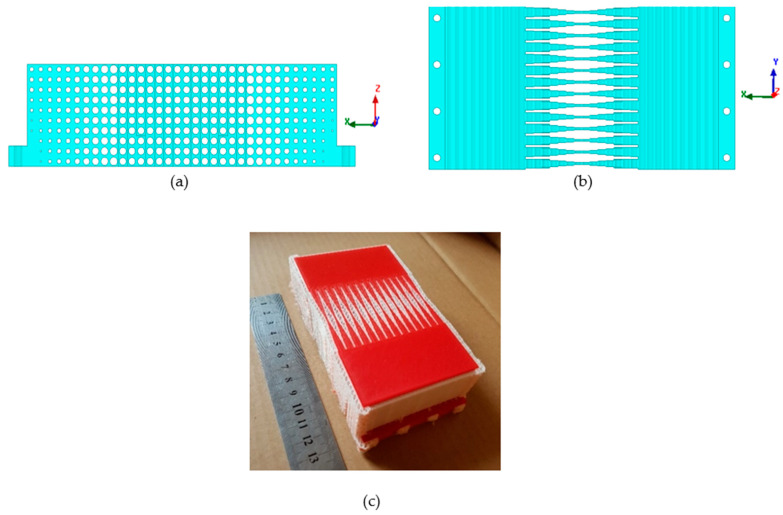
Realization of the DWTS. (**a**) Front view. (**b**) Top view. (**c**) Fabricated prototype.

The unit cells consisted of cylindrical air holes of different dimensions, as illustrated in Figure 3. Changing the radius ‘*r*’ and thickness ‘*t*’ of the dielectric part allowed us to tailor the effective permittivity value ranging from 1 to 2.65 of the unit cell while keeping the periodicity 5 mm along the *y*-axis, as shown in Figure 4.

The effective permittivity of the dielectric unit cells was numerically characterized using a parameter retrieval method [24]. This method is based on the extraction of the transmission and reflection coefficients from full-wave simulations of a unit cell with periodic boundary conditions. These coefficients are used to determine the wave impedance *Z* and the refractive index *n*, and they are based on values of the effective permittivity of the dielectric unit cell being evaluated. The commercial code HFSS was used to derive the *S*-parameters of the dielectric unit cells.

Additive manufacturing 3D printing technology was used to fabricate the prototype. The structure was printed in layers using a commercially available 3D printer, where the lowest possible layer resolution of 0.06 mm was used in order to avoid any variation in effective permittivity value. This way of printing enabled the realization of the DWTS prototype with air holes of extremely small radius. A PLA plastic filament, having a dielectric constant of 2.65 and loss tangent of 0.0062 at 3 GHz, was used during the printing process. In order to further ensure the accuracy of the printed structure, another material polyvinyl alcohol (PVA) was used to fill the air holes during the printing process, which was easily removed by exposing the structure to water. The fabricated prototype of the device is shown in Figure 4c.

### 3.4. Complete Antenna System

**Figure 5:**
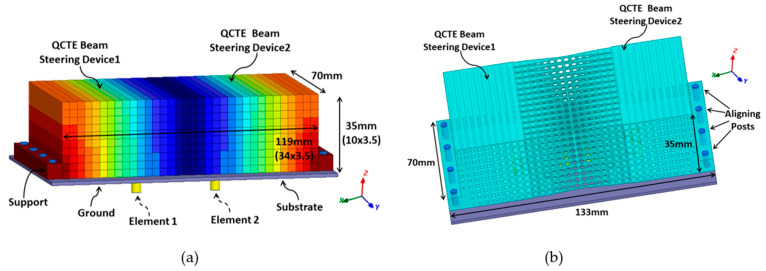
Geometry of the two-element MIMO antenna with the proposed DWTS. (**a**) Ideal DWTS. (**b**) PLA-based DWTS.

The proposed DWTS was placed above the patch-based two-element MIMO antenna, as shown in Figure 5. The dimensions of the ideal and PLA-based TE dielectric structure were 119 × 35 × 70 mm^3^. In order to mount the DWTS easily above the antenna elements, two supports of width 7 mm each with eight M3 holes were added at both edges. The structure was placed symmetrically above the two radiating elements such that its dielectric constant was 1 in the center and increased in steps moving on both sides along the *x*-axis with a maximum value of 2.65 at the edges. The structure was inserted symmetrically above the elements so that the branch power ratio remained close to 1, an attribute which is recommended for MIMO antennas.

## 4. Simulation and Measurement Results

### 4.1. S-Parameters and Radiation Patterns

**Figure 6:**
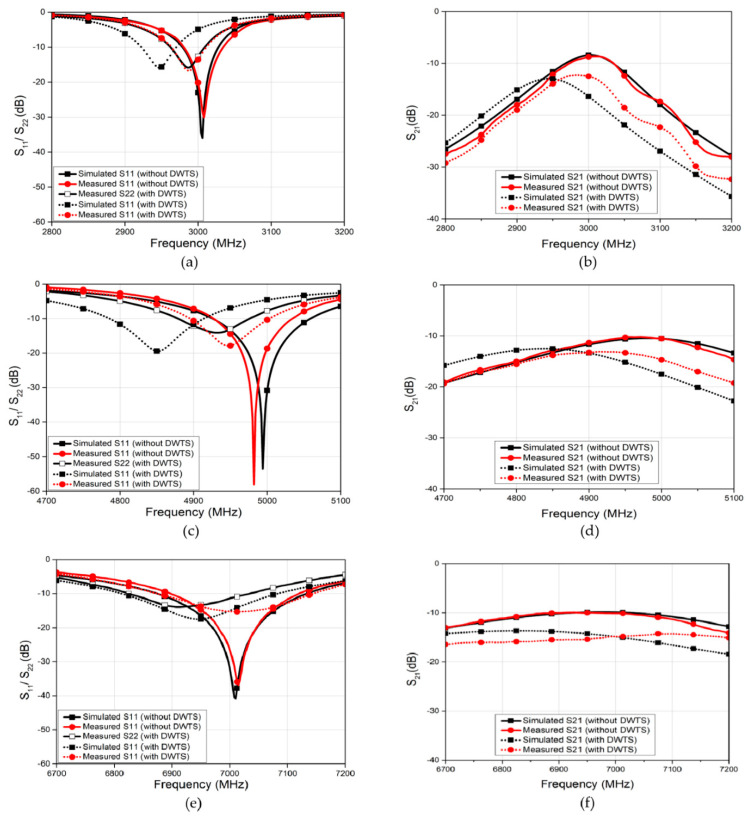
Simulated and measured S−parameters of the MIMO antenna system with and without DWTS. (**a**) Reflection coefficient at 3 GHz. (**b**) Isolation at 3 GHz. (**c**) Reflection coefficient at 5 GHz. (**d**) Isolation at 5 GHz. (**e**) Reflection coefficient at 7 GHz. (**f**) Isolation at 7 GHz.

The complete antenna structures with ideal and PLA-based DWTS were modeled and numerically optimized in HFSS. For brevity, Figure 6 shows only the computed *S*-parameters of the MIMO antenna with and without the PLA-based DWTS at three tested frequencies, since similar responses were obtained with ideal DWTS. At 3 GHz, the antenna without the DWTS had a resonant frequency of 3005 MHz, with a fractional bandwidth of 1.6%. The isolation between the antenna elements was 8.4 dB. The resonant frequency of the antenna shifted slightly lower when the DWTS was placed above the radiating elements. Both elements maintained the same −10 dB bandwidth with a resonant frequency of 2945 MHz. The DWTS improved the isolation between the closely placed antenna elements by 4.7 dB. At 5 GHz and 7 GHz, the antenna without the DWTS had a resonant frequency of 4995 MHz and 7010 MHz, with a fractional bandwidth of 2.6% and 3.7%, respectively. The isolation between the antenna elements was 10.4 dB and 9.9 dB. The placement of DWTS resulted in slight shifting of the antenna’s resonance frequency toward lower side. Both antenna elements, however, maintained the same −10 dB bandwidth with a resonant frequency of 4850 MHz and 6950 MHz. The use of DWTS also resulted in isolation improvement between the closely placed antenna elements by 2.5 dB and 4.4 dB, at 4850 MHz and 6950 MHz, respectively. This increase in isolation is an added advantage of this technique when compared to the PRS-based beam tilting described in [12]. This is mainly due to the beam tilting without forming a cavity since there is no significant reflection from the cavity walls to decrease the isolation.

**Figure 7:**
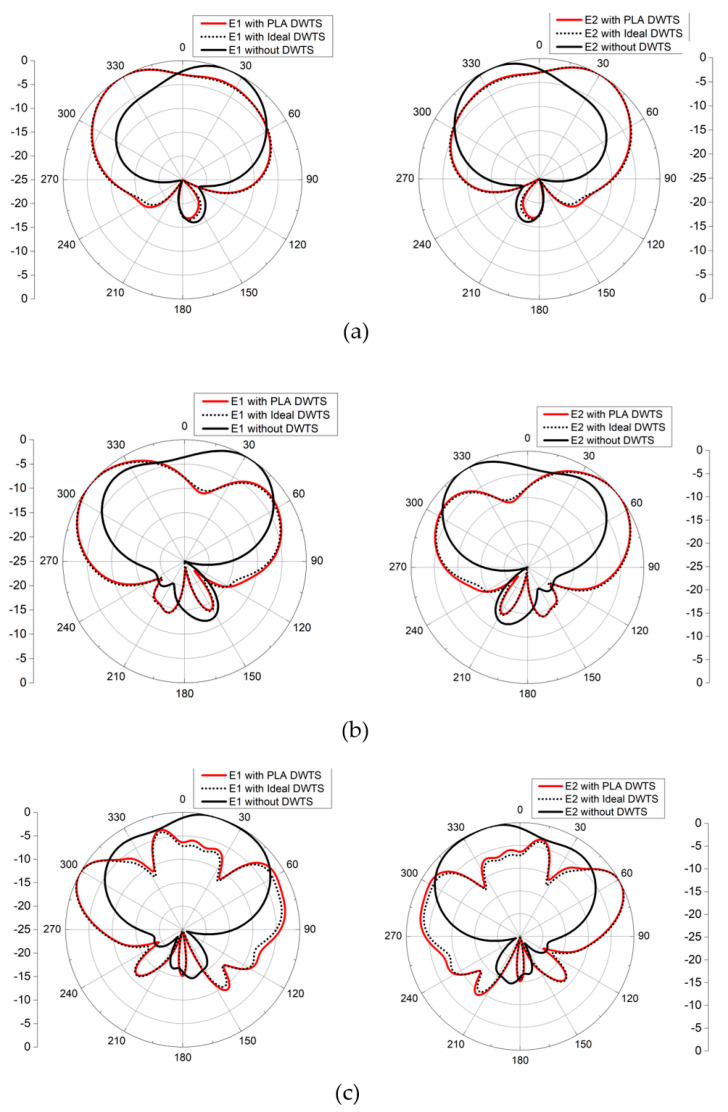
Simulated normalized 2D radiation pattern of MIMO antenna with and without the DWTS in *xoz*−plane. (**a**) E1 and E2 at 3 GHz. (**b**) E1 and E2 at 5 GHz. (**c**) E1 and E2 at 7 GHz.

**Figure 8:**
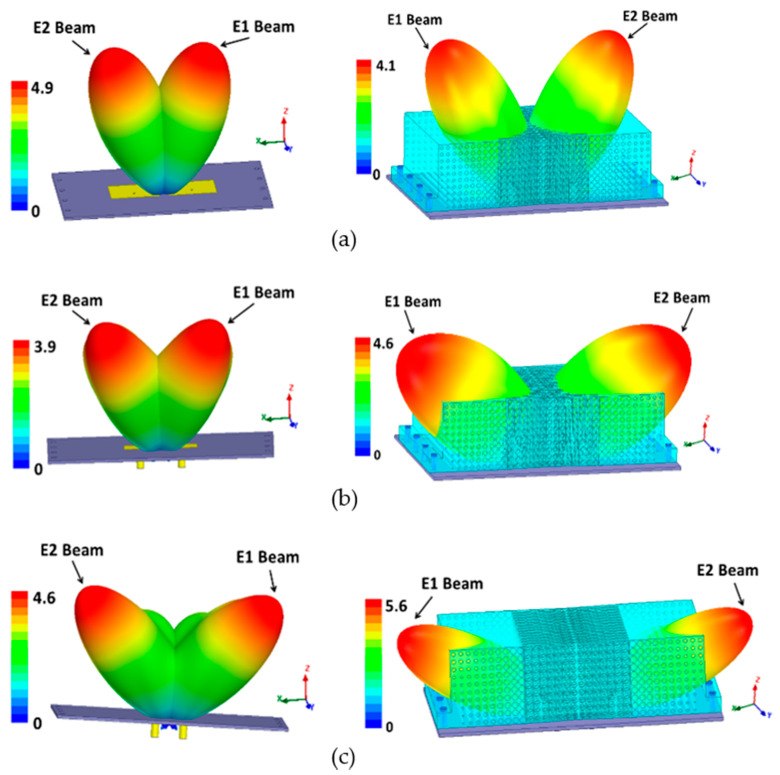
3D radiation pattern (in linear scale) of the two-element MIMO antenna with and without the DWTS. (**a**) 3 GHz. (**b**) 5 GHz. (**c**) 7 GHz.

The far-field simulations of the two-element MIMO antenna with and without the DWTS were analyzed at their three resonant frequencies. To obtain the radiation pattern, one element was excited while the other was terminated with 50 Ω matched load. Figure 7 shows the 2D normalized radiation patterns of the antenna in the *xoz*-plane for both cases. For the case of the MIMO antenna without the DWTS, each antenna element had a typical radiation pattern of a patch antenna, which was slightly tilted due to the presence of another element in close vicinity. It can be seen from the figure that the beam maxima of the antenna elements E1 and E2 were at 24°/32°/16° and −24°/−32°/−16°, respectively. By introducing the DWTS, the beam maxima of antenna elements E1 and E2 tilted to −31°/−50°/−62° and 31°/50°/62°, respectively. The proposed device, therefore, tilted the beam of each antenna element by 55°/82°/78°, thereby decreasing the overlap between the two beams. A 3D field visualization of the MIMO antenna elements without and with the proposed structure placed on the model at 3 GHz, 5 GHz, and 7 GHz is shown in Figure 8. The realized gain of each antenna element with the DWTS was 6.1 dB, 6.6 dB, and 7.5 dB at 3 GHz, 5 GHz, and 7 GHz, respectively. The radiation efficiency of each antenna element with the proposed device increased from 81.4% to 85.7% at 3 GHz and stayed the same at 5 GHz and 7 GHz. This improvement can be credited to the reduction in coupling between the two antenna ports.

**Figure 9:**
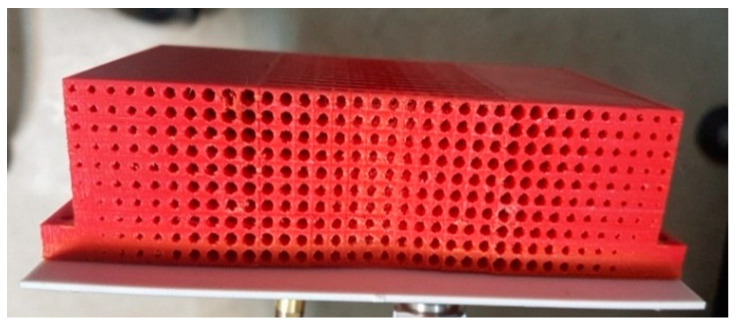
Printed DWTS mounted above MIMO antenna system.

The two-element rectangular patch-based MIMO antennas operating at 3 GHz, 5 GHz, and 7 GHz were printed on a Rogers substrate RO4003 using an LPKF milling machine. Plastic M3 screws were used for fastening the device on each antenna substrate so that there was minimal influence on the characteristics of the antenna. The fabricated antenna system with DWTS is shown in Figure 9. The *S*-parameters were measured to verify the performance of each antenna with and without the proposed structure. Both the simulated and the measured *S*-parameters at three tested frequencies are shown in Figure 6. The overall measured results are in good agreement with those obtained from simulations. In all three cases, the resonance of both antenna elements with the proposed DWTS was seen to shift slightly toward the lower frequencies, with *S*_11_ and *S*_22_ below the −10 dB level, having fractional bandwidths of 1.6%, 2.2%, and 3.8%, respectively. The shifted resonance frequency of both elements in each case was 2987 MHz, 4948 MHz/4932 MHz, and 7015/6912 MHz, respectively. In addition, the measured isolation level *S*_21_ between the two ports of closely spaced antenna elements improved by more than 3.9 dB, 3 dB, and 4.6 dB at each shifted resonance frequency, respectively.

**Figure 10:**
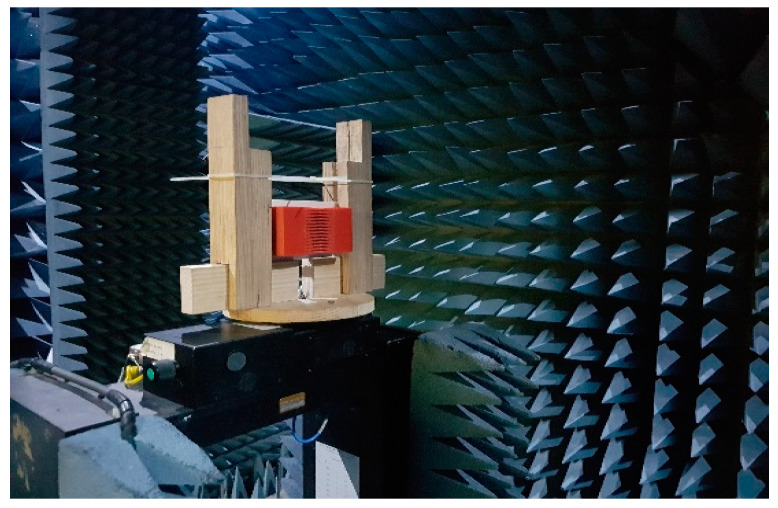
Fabricated antenna measurement in anechoic chamber.

**Figure 11:**
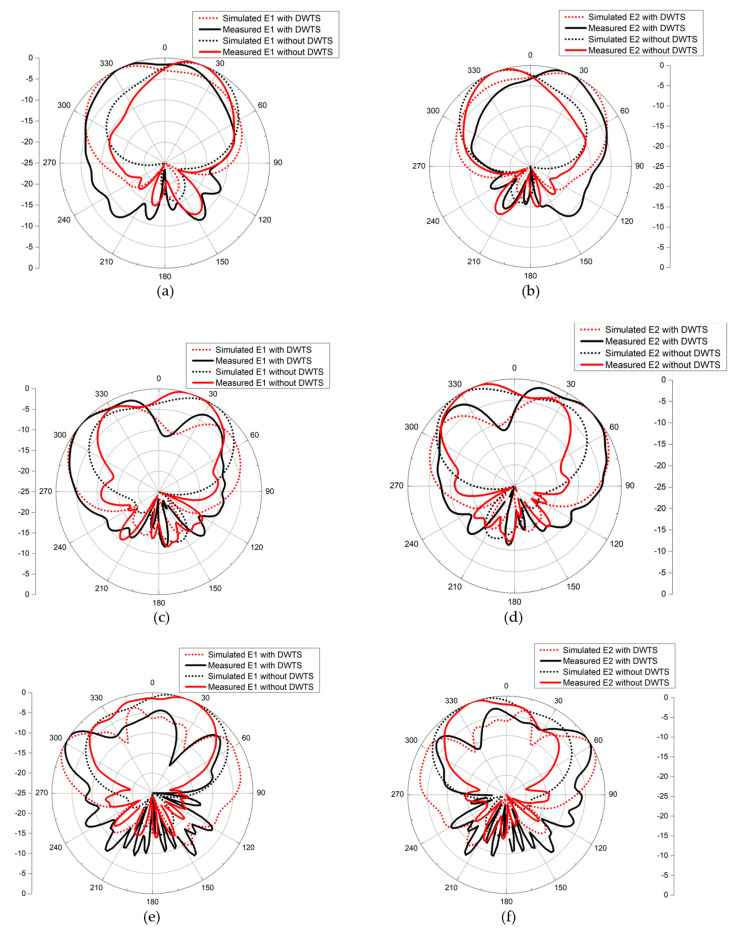
Simulated and measured radiation patterns (in the *xoz*−plane) for both configurations. (**a**) E1 at 3 GHz. (**b**) E2 at 3 GHz. (**c**) E1 at 5 GHz. (**d**) E2 at 5 GHz. (**e**) E1 at 7 GHz. (**f**) E2 at 7 GHz.

The far-field measurement anechoic chamber at the Research Institute for Microwave and Millimeter-Wave Studies (RIMMS) was used to measure the radiation patterns of the antenna with and without the proposed DWTS (Figure 10). The measured and simulated 2D normalized radiation patterns of both elements in the *xoz*-plane at their respective resonance frequencies are presented in Figure 11. They were obtained by exciting one element and terminating the other element with 50 Ω matched load. The measured beam maxima without DWTS were slightly tilted to 20°/18°/20° and −20°/−22°/−20°, whereas, with the DWTS, the measured beam maxima were at −24°/−49°/−56° and 24°/45°/56°, respectively. This indicates a total beam tilt angle of 44°/67°/76° for both elements, which was somewhat less than the tilt angle of 55°/82°/78° predicted by simulations.

Other than measurement errors, the slight change in beams tilt angle could be attributed to two reasons. First, a change in the gradient index resulted from the slight modifications of the geometrical parameters, i.e., radius and thickness, due to the fabrication tolerance [25]. This slight change in beam tilt angle due to geometrical variations was not accounted for in the simulations. The second factor which modified the steering performance was the discretization process of the continuous permittivity profile of the DWTS. At each shifted resonance frequency, the measured gains at beam maxima for elements 1 and 2 with the DWTS were 4.8 dB/5.9 dB/6.4 dB, and 5.1 dB/6.4 dB/6.9 dB, respectively.

### 4.2. Correlation Coefficient and Other MIMO Parameters

Lastly, we evaluated *ρ*, which is the key performance figure of merit of the MIMO antenna. This parameter is calculated using the 3D field-based formulation of Equation (2), shown below, which takes into account the 3D far-field radiation patterns of the antenna elements with their polarizations, as well as the relative phase between the fields [3].

(2) $$\rho =\frac{{\left|\iint_{4\pi }\left[\;\vec{F}1\left(\theta ,ø\right)\;*\;{\vec{F}}_{2}\left(\theta ,ø\right)\right]d\Omega \right|}^{}}{\sqrt{\iint_{4\pi }{\left|\;{\vec{F}}_{1}\left(\theta ,ø\right)\;\right|}^{2}d\Omega \;\iint_{4\pi }\left|\;{\vec{F}}_{2}\left(\theta ,ø\right)\;\right|²d\Omega }},$$

where F*_i_*(*ø*, *ϕ*) (*i* = 1,2) is the far-field complex 3D radiation field of the *i*-th antenna, when only the *i*-th element is excited, and ∗ is the Hermitian product.

**Figure 12:**
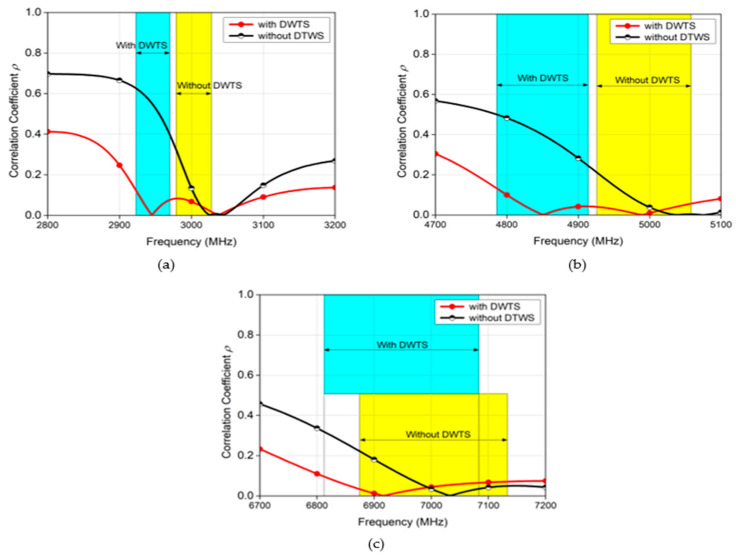
Correlation coefficient of the two-element MIMO antenna with and without the proposed DWTS. (**a**) 3 GHz. (**b**) 5 GHz. (**c**) 7 GHz.

Figure 12 shows the calculated *ρ* of the proposed antennas in their respective entire band of interest. It is evident from Figure 12 (blue and yellow highlighted regions that show the −10 dB bandwidth) that the *ρ* of the MIMO antenna presented higher values in the absence of the device than when the proposed DWTS was placed above the antenna elements. For the 3 GHz MIMO antenna, the maximum *ρ* value decreased from 0.3221 to 0.1233 at the −10 dB level, and from 0.0958 to 0.0000696 at the resonance point, without and with the DWTS device, respectively. On the other hand, for the 5 GHz/7 GHz MIMO antenna, the maximum *ρ* value decreased from 0.2119/0.2228 to 0.1322/0.0965 at the −10 dB level, and from 0.0465/0.02279 to 0.00159/0.00119 at each resonance point, without and with the DWTS device, respectively. This indicates that the radiation patterns tilting due to the proposed device placed above the MIMO antenna improved the field decorrelation because of reduced overlap between the radiation patterns, and the resulting reduction in *ρ* allowed achieving good diversity and multiplexing performances.

**Figure 13:**
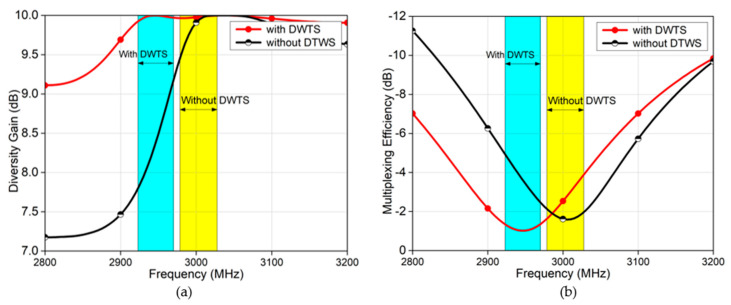
(**a**) Diversity gain of the two−element MIMO antenna with and without the proposed DWTS. (**b**) Multiplexing efficiency of the two-element MIMO antenna with and without the proposed DWTS.

Similarly, other performance parameters such as the diversity gain and multiplexing efficiency of the MIMO antenna elements, operating at 3 GHz, with and without DWTS, are shown in Figure 13. These parameters also indicated an improvement when DWTS was used with the antenna elements, reaffirming that correlation reduction by antenna beam tilting improves the MIMO diversity and multiplexing performance.

Although the beam tilt angle at 5 GHz and 7 GHz was large, which in an ideal case corresponds to a large reduction in *ρ*, the corresponding reduction in *ρ* was not that significant compared to the 3 GHz case. The variation in *ρ* values with frequency can be understood in relation to the radiation patterns of Figure 7a–c. At both resonant frequencies, the overlapping of relatively large sidelobes caused a degradation in the *ρ* value.

Table 1 compares the performance of the proposed technique with that of similar methods where beam tilts of MIMO antennas were used to reduce the correlation coefficient, showing that the proposed concept provides an improved performance over legacy designs. It is evident that the proposed broadband technique not only decreases the correlation coefficient, but also improves the isolation between the antenna elements, which in turn results in an improved performance over techniques that have been reported in the past for closely placed antenna elements.

## 5. Conclusions

This paper presented a new broadband method based on the use of a non-resonant all-dielectric device for simultaneously reducing the correlation coefficient and mutual coupling by decorrelating the radiation pattern of two closely spaced antenna elements in an MIMO configuration operating at three different frequencies. It is shown that field decorrelation can be achieved by tilting the far-field radiation patterns of the individual antenna elements away from each other by appropriately designing and placing the DWTS above the MIMO antenna elements. The effectiveness of the proposed technique was verified by comparing simulated and measured far-field results at three different frequencies, which achieved simultaneous improvement in the correlation coefficient and port isolation. Thus, it is shown that the proposed broadband solution realizes an improved MIMO diversity, as well as multiplexing performance. Furthermore, the proposed technique can be conveniently extended to systems which include more than two elements, as is the case in many real-world scenarios.

## Figures and Tables

**Table 1:** Comparison of proposed method with previous reported works.

Ref.	Radiating Element/Ports	Edge to Edge Spacing	Beam Tilt Angle	Isolation	Reduction in Correlation
[12]	Patch/2	0.13λ	51°	Degrade	95%
[26]	DRA/6	0.31λ	45°	Improve	42.3%
[27]	DRA/4	0.24λ	35°	Improve	43.2%
[28]	Patch/4	0.23λ	27°	Degrade	65%
[29]	Patch/2	0.03λ	35°	Improve	Not given
[30]	Patch/2	0.15λ	0°	Improve	Inaccurate
[31]	Patch/2	0.17λ	0°	Improve	Not given
This work	Patch/2	0.06λ	44°	Improve	62% to 99%
		at 3 GHz			
		0.1λ	67°		37% to 97%
		at 5 GHz			
		0.1λ	76°		57% to 95%
		at 7 GHz			

## Data Availability

Not applicable.

## References

[1] Paulraj A.J., Gore D.A., Nabar R.U., Bölcskei H. (2004). An overview of MIMO communications-A key to gigabit wireless. Proc. IEEE.

[2] Costa J.R., Lima E.B., Medeiros C.R., Fernandes C.A. (2011). Evaluation of a new wideband slot array for MIMO performance enhancement in indoor WLANs. IEEE Trans. Antennas Propag..

[3] Vaughan R.G., Andersen J.B. (1987). Antenna diversity in mobile communications. IEEE Trans. Veh. Technol..

[4] Blanch S., Romeu J., Corbella I. (2003). Exact representation of antenna system diversity performance from input parameter description. Electron. Lett..

[5] Alibakhshikenari M., Virdee B.S., Shukla P., See C.H., Abd-Alhameed R., Khalily M., Falcone F., Limiti E. (2018). Antenna Mutual Coupling Suppression Over Wideband Using Embedded Periphery Slot for Antenna Arrays. Electronics.

[6] Yon H., Rahman N.H.A., Aris M.A., Jamaluddin M.H., Kong Cheh Lin I., Jumaat H., Mohd Redzwan F.N., Yamada Y. (2021). Development of C-Shaped Parasitic MIMO Antennas for Mutual Coupling Reduction. Electronics.

[7] Khan A., Geng S., Zhao X., Shah Z., Jan M.U., Abdelbaky M.A. (2020). Design of MIMO Antenna with an Enhanced Isolation Technique. Electronics.

[8] Elwi T.A. (2018). A miniaturized folded antenna array for MIMO applications. Wirel. Pers. Commun..

[9] Al-Dulaimi Z., Elwi T.A., Atilla D.C. (2020). Design of a Meander Line Monopole Antenna Array Based Hilbert-Shaped Reject Band Structure for MIMO Applications. IETE J. Res..

[10] Sharawi M.S. (2017). Current Misuses and Future Prospects for Printed Multiple-Input, Multiple-Output Antenna Systems [Wireless Corner]. IEEE Antennas Propag. Mag..

[11] Mikki S.M., Antar Y.M.M. (2015). On cross correlation in antenna arrays with applications to spatial diversity and MIMO systems. IEEE Trans. Antennas Propag..

[12] Hassan T., Khan M.U., Attia H., Sharawi M.S. (2018). An FSS Based Correlation Reduction Technique for MIMO Antennas. IEEE Trans. Antennas Propag..

[13] Qureshi U., Khan M.U., Hassan T., Sharawi M.S., Burokur S.N., Mittra R. Field decorrelation in a MIMO antenna using transformation electromagnetics. Proceedings of the 2020 International Workshop on Antenna Technology (iWAT).

[14] Pendry J.B., Schurig D., Smith D.R. (2006). Controlling electromagnetic fields. Science.

[15] Schurig D., Pendry J.B., Smith D.R. (2006). Calculation of material properties and ray tracing in transformation media. Opt. Express.

[16] Rahm M., Roberts D.A., Pendry J.B., Smith D.R. (2008). Transformation-optical design of adaptive beam bends and beam expanders. Opt. Express.

[17] Kwon D.H., Werner D.H. (2010). Transformation electromagnetics: An overview of the theory and applications. IEEE Antennas Propag. Mag..

[18] Hu J., Zhou X., Hu G. (2009). Design method for electromagnetic cloak with arbitrary shapes based on Laplace’s equation: Erratum. Opt. Express.

[19] Ma H.F., Cui T.J. (2010). Three-dimensional broadband and broad-angle transformation-optics lens. Nat. Commun..

[20] Tang W., Argyropoulos C., Kallos E., Song W., Hao Y. (2010). Discrete coordinate transformation for designing all-dielectric flat antennas. IEEE Trans. Antennas Propag..

[21] Yi J., Burokur S.N., de Lustrac A. (2015). Conceptual design of a beam steering lens through transformation electromagnetics. Opt. Express.

[22] Yi J., Burokur S.N., Piau G.P., de Lustrac A. (2016). Coherent beam control with an all-dielectric transformation optics based lens. Sci. Rep..

[23] Yi J., Burokur S.N., Piau G.P., de Lustrac A. (2016). 3D printed broadband transformation optics based all-dielectric microwave lenses. J. Opt..

[24] Smith D.R., Schultz S., Markoš P., Soukoulis C.M. (2002). Determination of effective permittivity and permeability of metamaterials from reflection and transmission coefficients. Phys. Rev. B-Condens. Matter Mater. Phys..

[25] Ratni B., Yi J., Ding X., de Lustrac A., Zhang K., Piau G.-P., Burokur S.N. (2018). Gradient phase partially reflecting surfaces for beam steering in microwave antennas. Opt. Express.

[26] Das G., Sharma A., Gangwar R.K., Sharawi M.S. (2019). Performance improvement of multiband MIMO dielectric resonator antenna system with a partially reflecting surface. IEEE Antennas Wirel. Propag. Lett..

[27] Das G., Sahu N.K., Sharma A., Gangwar R.K., Sharawi M.S. (2019). FSS-Based Spatially Decoupled Back-to-Back Four-Port MIMO DRA with Multidirectional Pattern Diversity. IEEE Antennas Wirel. Propag. Lett..

[28] Hassan T., Khan M.U., Shoaib N., Hussain R., Sharawi M.S. Correlation Reduction in a 4-Element MIMO Antenna using Partially Reflective Surface. Proceedings of the 13th European Conference on Antennas and Propagation (EuCAP).

[29] Qi H., Liu L., Yin X., Zhao H., Kulesza W.J. (2016). Mutual Coupling Suppression Between Two Closely Spaced Microstrip Antennas with an Asymmetrical Coplanar Strip Wall. IEEE Antennas Wirel. Propag. Lett..

[30] Wei K., Li J., Wang L., Xing Z., Xu R. (2016). Mutual Coupling Reduction by Novel Fractal Defected Ground Structure Bandgap Filter. IEEE Trans. Antennas Propag..

[31] Cheng Y., Ding X., Shao W., Wang B. (2017). Reduction of Mutual Coupling Between Patch Antennas Using a Polarization-Conversion Isolator. IEEE Antennas Wirel. Propag. Lett..

